# Supplementary material for: Splice-Junction-Based Mapping of Alternative Isoforms in the Human Proteome
Source: Cell Rep. Author manuscript; Available in PMC 2020 Jan 15. (PMC6961840; doi:10.1016/j.celrep.2019.11.026)
Supplement: 2 [file NIHMS1546469-supplement-2.zip › DF1/PXD010154/Heart/Heart_41_TTN_ASNEYGSVSCTATLTVTEPPSFIKK.pdf]

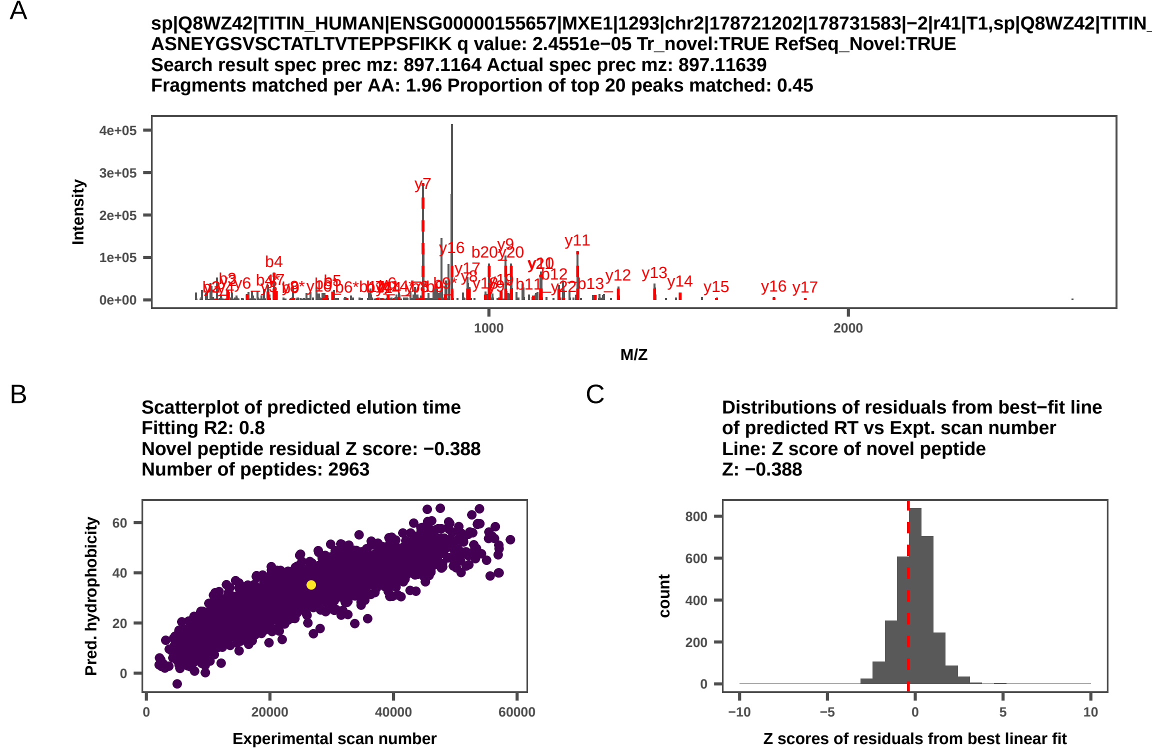
A
sp|Q8WZ42|TITIN_HUMAN|ENSG00000155657|MXE1|1293|chr2|178721202|178731583|−2|r41|T1,sp|Q8WZ42|TITIN_
ASNEYGSVSCTATLTVTEPPSFIKK q value: 2.4551e−05 Tr_novel:TRUE RefSeq_Novel:TRUE
Search result spec prec mz: 897.1164 Actual spec prec mz: 897.11639
Fragments matched per AA: 1.96 Proportion of top 20 peaks matched: 0.45
Intensity
4e+05
3e+05
2e+05
1e+05
0e+00
y7
y16
b20
y9
y20
y11
b4
y17
y8
y10
b12
y12
y13
y14
y15
y16
y17
b5
b11
b13
1000
2000
M/Z
B
Scatterplot of predicted elution time
Fitting R2: 0.8
Novel peptide residual Z score: −0.388
Number of peptides: 2963
Pred. hydrophobicity
60
40
20
0
0
20000
40000
60000
Experimental scan number
C
Distributions of residuals from best−fit line
of predicted RT vs Expt. scan number
Line: Z score of novel peptide
Z: −0.388
count
800
600
400
200
0
−10
−5
0
5
10
Z scores of residuals from best linear fit
